# Automated lesion detection on MRI scans using combined unsupervised and supervised methods

**DOI:** 10.1186/s12880-015-0092-x

**Published:** 2015-10-30

**Authors:** Dazhou Guo, Julius Fridriksson, Paul Fillmore, Christopher Rorden, Hongkai Yu, Kang Zheng, Song Wang

**Affiliations:** Department of Computer Science & Engineering, University of South Carolina, 301 Main Street, Columbia, 29201 USA; Department of Communication Science & Disorders, University of South Carolina, 915 Greene Street, Columbia, 29208 USA

**Keywords:** Lesion detection, Magnetic resonance imaging (MRI), Unsupervised and supervised methods

## Abstract

**Background:**

Accurate and precise detection of brain lesions on MR images (MRI) is paramount for accurately relating lesion location to impaired behavior. In this paper, we present a novel method to automatically detect brain lesions from a T1-weighted 3D MRI. The proposed method combines the advantages of both unsupervised and supervised methods.

**Methods:**

First, unsupervised methods perform a unified segmentation normalization to warp images from the native space into a standard space and to generate probability maps for different tissue types, e.g., gray matter, white matter and fluid. This allows us to construct an initial lesion probability map by comparing the normalized MRI to healthy control subjects. Then, we perform non-rigid and reversible atlas-based registration to refine the probability maps of gray matter, white matter, external CSF, ventricle, and lesions. These probability maps are combined with the normalized MRI to construct three types of features, with which we use supervised methods to train three support vector machine (SVM) classifiers for a combined classifier. Finally, the combined classifier is used to accomplish lesion detection.

**Results:**

We tested this method using T1-weighted MRIs from 60 in-house stroke patients. Using leave-one-out cross validation, the proposed method can achieve an average Dice coefficient of 73.1 *%* when compared to lesion maps hand-delineated by trained neurologists. Furthermore, we tested the proposed method on the T1-weighted MRIs in the MICCAI BRATS 2012 dataset. The proposed method can achieve an average Dice coefficient of 66.5 *%* in comparison to the expert annotated tumor maps provided in MICCAI BRATS 2012 dataset. In addition, on these two test datasets, the proposed method shows competitive performance to three state-of-the-art methods, including Stamatakis et al., Seghier et al., and Sanjuan et al.

**Conclusions:**

In this paper, we introduced a novel automated procedure for lesion detection from T1-weighted MRIs by combining both an unsupervised and a supervised component. In the unsupervised component, we proposed a method to identify lesioned hemisphere to help normalize the patient MRI with lesions and initialize/refine a lesion probability map. In the supervised component, we extracted three different-order statistical features from both the tissue/lesion probability maps obtained from the unsupervised component and the original MRI intensity. Three support vector machine classifiers are then trained for the three features respectively and combined for final voxel-based lesion classification.

## Background

Accurate detection of lesions in the brain is critical to both clinical practice and neuropsychological research. For example, every year more than 795,000 people in the United States suffer a new or recurrent stroke (http://www.strokeassociation.org) and the identification and analysis of the brain lesions resulting from a stroke can help understand the lesion-deficit relationship [1, 2], predict patient diagnosis and prognosis [3], and chart the development of brain pathology over time [3]. In the past two decades, Magnetic Resonance Imaging (MRI) has become a reliable and increasingly popular technique for identifying brain damage and pathologies [4]. To study brain lesions using MRI, the first step is to accurately detect the lesion from different-modality MRIs (e.g. Diffusion-weighted imaging, T1-MRI, FLAIR, or T2-MRI). Here we focus on T1-weighted images in chronic stroke, as this modality is generally available, provides high spatial resolution and good contrast between gray matter (GM), white matter (WM) and cerebral spinal fluid (CSF). The goal of the research presented here is to develop a fully automatic algorithm for accurately detecting lesions from T1-MRI scans in chronic stroke patients.

Traditionally, the gold standard for lesion detection relied on manual delineation by one or more trained neurologists/radiologists creating a binary lesion mask [5]. These methods show high reliability (e.g. 0.86–0.95) between raters [4, 6]. However, manual labeling is laborious and subjective. In recent years, several automated methods have been developed for brain lesion detection [7–13]. However, automated brain lesion detection from MR images is still a very challenging problem, particularly when only the T1-MRI is available. First, it is sometimes difficult to separate the lesion from the surrounding tissues that is relatively structurally intact based only on image intensity since the intensities of the lesion and healthy tissues may be similar, not to mention that the intensity of the lesion may not be homogeneous. Second, lesions are often non-rigid and complex in shape and vary greatly in size and position across different patients. Therefore it is difficult to construct a compact and informative geometric ‘prior’ to guide lesion detection.

In general, existing automatic lesion detection methods can be divided into two categories: (1) unsupervised methods based on prior knowledge [9–21]; (2) supervised methods based on machine learning [7, 22–29].

For unsupervised methods, general knowledge or assumptions (*prior*s) derived from healthy controls, such as spatial locations of different brain tissues, are used to guide the brain segmentation and the lesion detection – the presence of a lesion usually breaks such general priors derived from healthy controls. In [13], a statistical method based on the Markov random field is developed to identify the lesion by comparing the image of a patient and the images of a group of healthy controls. Shen et al. [18] found that, in the lesion regions, the intensity-based segmentation and the location of the tissues are inconsistent with these in healthy controls. They use a fuzzy c-means algorithm to quantify such inconsistencies and then apply a threshold to obtain a binary lesion segmentation. This method was further improved in [19] by introducing 1) a normalized inconsistency measurement, which enables the use of a better threshold, and 2) an extra step that can better distinguish the ventricles from the lesions. Seghier et al. [11] extend a widely used brain-segmentation algorithm [30] to handle lesion detection by introducing a new tissue class for lesions. This extended segmentation algorithm iteratively performs nonrigid atlas-based registration to refine the probability maps of gray matter (GM), white matter (WM), cerebrospinal fluid (CSF), and subject-specific lesions. After that, the refined probability maps of GM and WM are fed into a fuzzy clustering procedure [31] for final lesion classification. This algorithm was further improved by Sanjuan et al. [20] by introducing a new procedure for updating the probability map of subject-specific lesions. Without using lesion samples manually annotated by experts, these unsupervised methods may not accurately capture the subtle difference between the lesion and its surrounding healthy tissues.

For supervised methods, a set of (training) image samples, on which lesions have been manually annotated by experts, are used to train a classifier, which can then be used for lesion detection on a new image by classifying each voxel on this image as lesion or healthy tissue. In Anbeek et al. [7], a K-Nearest Neighbor (KNN) classifier is trained for white matter lesion detection by combining intensity information from T1-weighted, T2-weighted, proton density-weighted (PD), and fluid attenuation inversion recovery (FLAIR) MR scans. Based on the same features, Lao et al. [26] suggest the use of support vector machine (SVM) instead of KNN to improve the accuracy of white matter lesion detection. Quddus et al. [28] combine SVM with a boosting technique to detect lesions in PD scans. Morra et al. [27] train a SVM classifier for multiple sclerosis (MS) lesion detection by using Haar-like wavelet features, which are extracted from T1-weighted, T2-weighted, FLAIR, mean diffusivity, and fractional anisotropy MR scans. Schneell et al. [29] extract voxel-based features by measuring each voxel’s likelihood to be located in the five selected brain areas and the background from a high angular resolution diffusion imaging (HARDI) scan. Based on these features, a SVM classifier is then trained to distinguish voxels from lesion and those from healthy tissues. Geremia et al. [25] propose a random decision forest method [32] for MS lesion detection by using local and context-rich features [33] from T1-weighted, T2-weighted and FLAIR MR scans. Jiang et al. [34] extract Gabor features [35] from multimodal MRIs and then use a distance metric learning [36] to train a classifier to classify each voxel, followed by a graph-cut operation for final segmentation of tumors. To achieve accurate lesion detection, these supervised methods usually require the use of multimodal MRIs, especially the T2-weighted, PD and FLAIR MRIs on which the lesion and healthy tissues show higher intensity difference than on the T1-weighted MRIs [37, 38].

In this paper, we propose a new lesion detection method based only on T1-weighted MR images by combining the advantages of unsupervised and supervised methods. Specifically, we first conduct a modified enantiomorphic normalization [39] to warp the native T1-weighted MRI into the MNI standard space [40] and construct an initial lesion probability map (LPM) by comparing the normalized MR image with the healthy controls. Then we perform non-rigid and reversible atlas-based registration to refine the template of gray matter probability map, the template of white matter probability map, the template of external CSF probability map, the template of ventricle probability and initial LPM. These probability maps are combined with the original MR image intensity to construct high-dimensional voxel-based features, with which we supervisedly train a combined SVM classifier for final lesion detection. The unsupervised lesion probability map endues more anatomic/spatial information to the voxel-based features, which increases the discriminations of the lesion and the healthy tissues on the T1-MRIs. For the final supervised lesion detection, we formulate the problem as a feature-set classification where different SVM classifiers are learned for different features. Using the leave-one-out cross-validation, the proposed method can achieve an average Dice coefficient of 73.1 *%* on in-house stroke patients and an average Dice coefficient of 66.5 *%* on MICCAI BRATS 2012 dataset, when compared to expert annotated lesion maps.

## Methods

### Study design

This study adhered to human experimentation guidelines of the U.S. Department of Health and Human Services and Helsinki Declaration. The CDC Institutional Review Board approved the study protocols (IRB # Pro00005458). All participants were volunteers who gave a written and verbal consent to be included in the study and were not required to give informed consent to allow us to show the images in published figures.

### Method overview

**Fig. 1:**
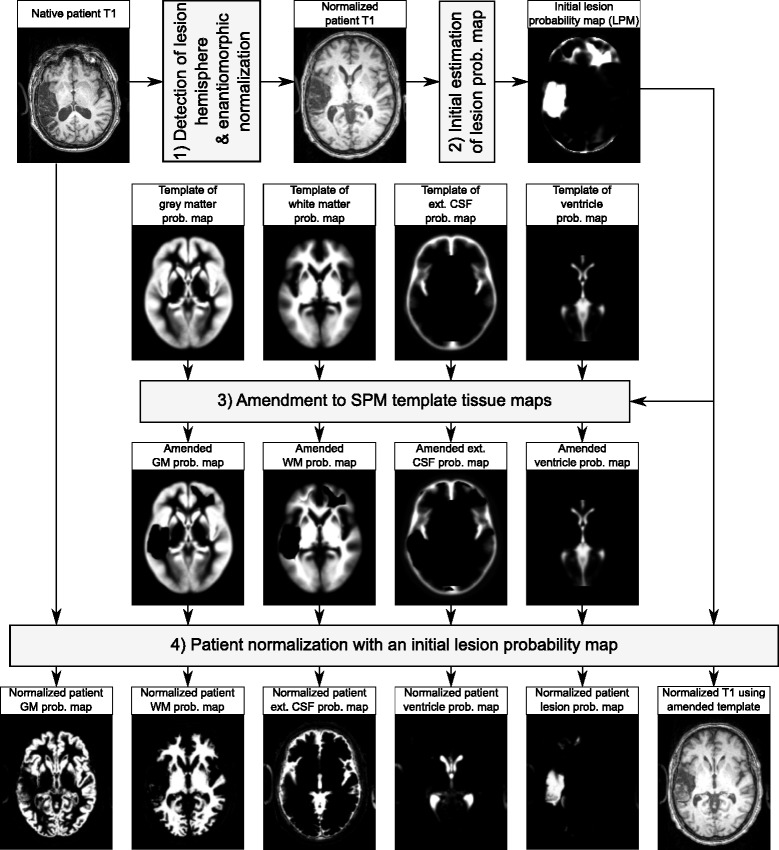
An illustration of the unsupervised component

The proposed method detects lesions on T1-weighed MR images by two sequential components: an *unsupervised* component to construct an LPM, followed by a *supervised* component to detect the final lesion areas. As shown in Fig. 1, the unsupervised component consists of four sequential steps: 1) detection of lesion-hemisphere and enantiomorphic normalization that normalizes the input T1-weighted MRI into a standard space, 2) comparing the normalized MRI to a group of normalized healthy controls and identifying an *initial* probability map of the lesion (LPM) by using the fuzzy clustering pipeline (FCP), 3) amending the template probability maps of GM, WM, external CSF and ventricle using the *initial* LPM, and 4) refining the LPM and constructing the probability maps of GM, WM, external CSF and ventricle from the input (normalized) MRI by mapping to the amended template [30]. Note that, in Step 3) a new amended template, including four tissue probability maps, needs to be constructed for each input T1-weighted MRI separately.

**Fig. 2:**
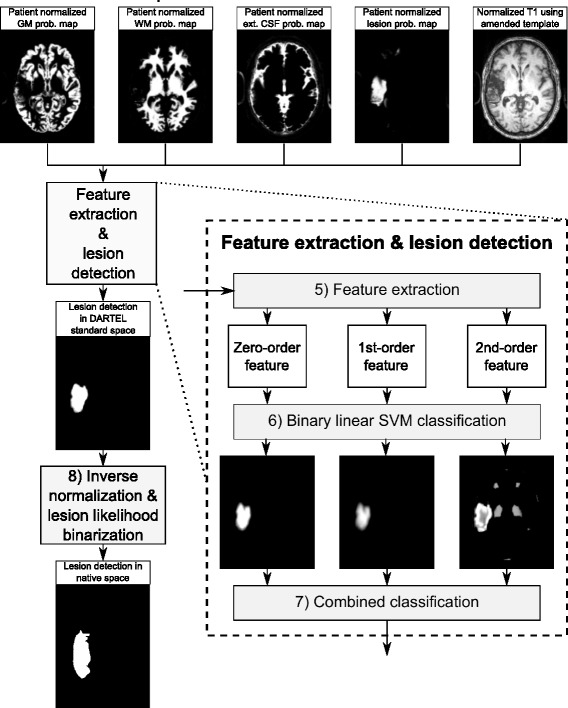
An illustration of the supervised component

As shown in Fig. 2, the supervised component also consists of four sequential steps: 5) at each voxel, extracting zero-order, 1st-order, and 2nd-order statistical features from the probability maps of GM, WM and external CSF and the refined LPM resulted from the unsupervised component, and the original T1-weighted MRI; 6) training three binary SVM classifiers that recognize each voxel to be part of lesion or not, by using the three types of statistical features, respectively; 7) combining the three SVM classifiers into a single SVM classifier and applying the combined SVM classifier to detect the lesion voxels; and 8) performing inverse normalization to get the lesions in the original T1-weighted MRI. In this paper, we normalize the brain to the MNI standard space for lesion estimation, feature extraction and final lesion classification to make the parameter setting of proposed method insensitive to the different-size brains and different-resolution images.

Besides the basic idea of combining the unsupervised and supervised components, we also make technical contributions in several of the steps. In Step 1), we automatically detect the lesion hemisphere and only use the healthy hemisphere for enantiomorphic normalization. This step improves the accuracies of enantiomorphic normalization and the final lesion detection. We will discuss it in detail in Section “Step 1): Lesion hemisphere detection and enantiomorphic Normalization”. In Step 2), we extend the FCP [31] to construct the initial LPM. This step will be briefly discussed in Section “Step 2): Initial estimation of LPM”. In Step 3), we amend the template probability maps of the four brain tissues and refine the initial LPM, which will be elaborated in Section “Step 3): Amendment to normalization-segmentation template tissue maps”. From Step 5) to Step 7), we apply statistical feature based supervised learning algorithm to further improve the lesion detection accuracy. We briefly discuss this algorithm in Section “Step 5): Feature extraction”. In this paper, Step 4) simply follows the six-tissue unified segmentation normalization (USN) pipeline [30] in SPM8 [41] (‘new segment’ in SPM8, referred to as ‘segment’ in SPM12) and Step 8) first follows the inverse normalization in SPM8 [41] and then binarizes the lesion likelihood map into a binary lesion mask.

### Unsupervised component

As mentioned in Section “Method overview”, in the unsupervised components, we focus on Step 1) detecting the lesioned hemisphere and enantiomorphic normalization, Step 2) constructing initial LPM using FCP and Step 3) amending the template tissue probability maps and refining the initial LPM.

#### Step 1): Lesion hemisphere detection and enantiomorphic Normalization

To spatially align the brains of a patient and a set of healthy controls and make them comparable, we normalize all the brain MRIs (i.e., both the patient and the healthy controls) to the MNI standard space. For the healthy controls, we can use the USN pipeline [30] in SPM8 [41] for this purpose. USN is a probabilistic framework based on a mixture of Gaussian distributions that enables nonlinear image registration, tissue classification,and bias correction. However, note that the patient’s T1 scan has an unusual appearance at the location of the lesion (the feature we are trying to detect), while the template images used for normalization are based on healthy individuals. The difference in intensity between the lesion and corresponding locations in the template can disrupt the automated normalization process [42]. This step is inspired by the previously developed enantiomorphic normalization procedure [39], where the lesion region is manually delineated and replaced by its mirror region from the counter hemisphere prior to normalization. Because the lesion region is not priorly known here, we estimate the healthy brain underlying a lesioned brain by first identifying the healthy hemisphere and then using its mirror copy to replace the lesioned hemisphere.

It is important to note that brains are by nature asymmetric, even the healthy brains are not perfectly symmetric [43]. Without knowing the ground-truth lesion mask, here we assume the brain symmetry to construct a rough estimate of the healthy brain underlying a lesioned brain, for enantiomorphic brain normalization to the standard space. While we know both the estimated healthy brain and the enantiomorphic normalization are not perfect, we only use them to estimate an initial LPM and expect that the later steps will further refine the initial LPM. In Section “Discussion”, we conduct experiment to analyze the impact of the brain asymmetry to the proposed method.

**Fig. 3:**
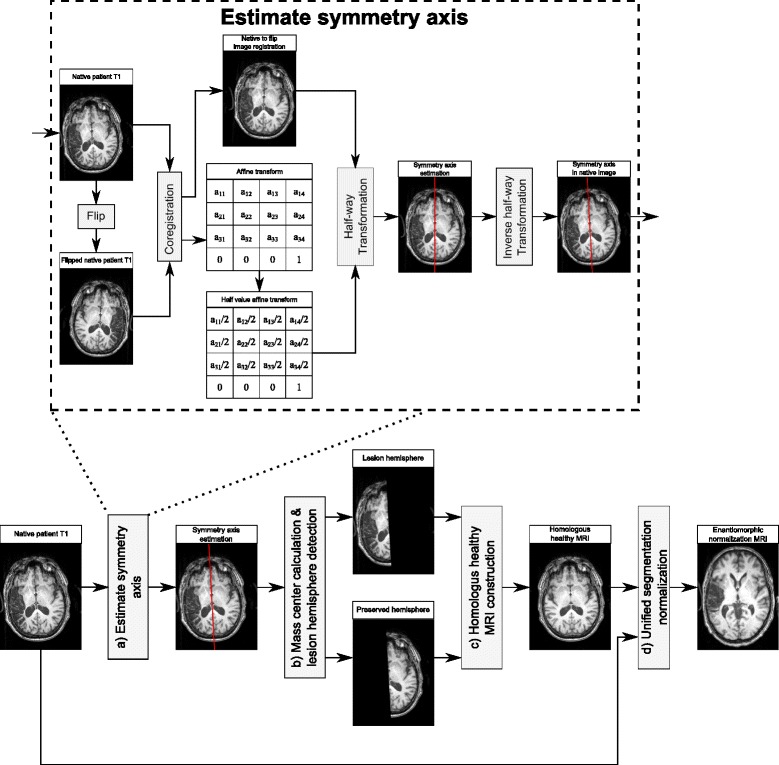
An illustration of the symmetry axis estimation and enantiomorphic normalization

As illustrated in Fig. 3, the proposed normalization procedure consists of the following steps.

a) Estimate the symmetry axis/plane, i.e., the middle plane that separates the two hemispheres, as shown by the red line in Fig. 3. In this paper, we use the tool developed by Rorden [44] for the symmetry axis estimation. Specifically, let *I*_*s*_ be the source MRI of the patient, with size *x*_*max*_×*y*_*max*_×*z*_*max*_, and we construct a reference image *I*_*r*_ by flipping *I*_*s*_ over the *x*−*z* plane, i.e., *I*_*r*_(*x,y,z*)=*I*_*s*_(*x,y*_*max*_−*y,z*) for all the voxels (*x,y,z*). We then use SPM8 coregistration routine [41, 45] to rigidly register *I*_*s*_ and *I*_*r*_ using an affine transformation *T*_*sr*_, i.e., *T*_*sr*_(*I*_*s*_) is spatially aligned with *I*_*r*_. This registration is achieved by maximizing the mutual information metric. By applying half of this transformation to the source image, we construct a half-way image, as illustrated in Fig. 3. $I_{h}=\frac {1}{2}T_{\textit {sr}}(I_{s})$ and the symmetry axis of *I*_*h*_ is the plane $y=\frac {1}{2}y_{\textit {max}}$. This plane can be mapped to the source image by using the inverse transform $2T_{\textit {sr}}^{-1}$, which is the desired symmetry axis in the source image, as shown by the red line in Fig. 3.

b) Identify the lesioned hemisphere. Since the intensity of a lesion on T1-MRI is usually lower than its counter region in the other hemisphere without a lesion, we calculate the intensity-weighted center of mass of *I*_*s*_ as

(1) $$\begin{array}{@{}rcl@{}}  \frac{\sum_{(x,y,z)}(x,y,z)\cdot I_{s}(x,y,z)}{\sum_{(x,y,z)}I_{s}(x,y,z)}. \end{array} $$

This center of mass would be biased towards the healthy hemisphere. Therefore, by assuming that the lesioned hemisphere is darker than the healthy hemisphere we can detect the lesioned hemisphere. Note that this step could be modified to process other modality MRIs. For example, in the T2-weighted MRIs, lesion regions tend to show higher intensities than the healthy tissues.

c) Estimate the underlying healthy brain. This can be achieved by replacing the lesioned hemisphere using the mirror of the healthy hemisphere, flipped over the symmetry axis.

d) Normalize the patient MRI. We first use the nonlinear USN in SPM8 to warp the estimated healthy brain to the MNI standard space and then apply the same transformation to the native patient image for normalization.

#### Step 2): Initial estimation of LPM

In this step, our goal is to construct an initial lesion probability map (LPM) based on the normalized patient image. In the standard space, we compare the intensity of each voxel between the patient and the healthy controls to estimate the lesion likelihood of the voxel in the patient image. For each brain T1-MRI, we take the healthy hemisphere for estimating the intensity distribution and then normalize this distribution [1, 46, 47] to calculate the Z-score for all the voxels in the brain.

Similarly, each healthy control T1-MRI is converted to Z-scores based on the whole brain mean and standard deviation. To suppress image noise, we further perform a Gaussian smoothing, with a kernel of 8 *mm* full-width-half-maximum (FWHM), to each image [13, 48]. Let $\hat {I_{s}}(x,y,z)$ and $\overline {I}_{h}(x,y,z)$ be the intensity of the patient image and the average intensity over all the healthy controls at voxel (*x,y,z*) – be reminded that both patient image and healthy-control images have been normalized to the standard space and their intensities have been unified to z-scores.

First, we calculate the dissimilarity (distance in intensities) [31] between the patient and healthy controls at voxel (*x,y,z*) by

(2) $$\begin{array}{@{}rcl@{}} \Delta I(x,y,z)=tanh\left(\frac{\hat{I}_{s}(x,y,z)-\overline{I}_{h}(x,y,z)}{\alpha}\right), \end{array} $$

where *α* controls the sensitivity in detecting the lesion voxels. As mentioned in Step 1): Lesion hemisphere detection and enantiomorphic Normalization Step b), on T1-weighted MRIs, the intensity of lesions is usually lower compared to the intensity of the healthy tissues at the mirrored locations across the symmetry axis. This way, it is expected that the lesion voxels show negative *ΔI*(*x,y,z*). We therefore define an initial lesion probability *P*_*L*_(*x,y,z*) as

(3) $$\begin{array}{@{}rcl@{}} P_{L}(x,y,z)=\left\{ \begin{array}{cc} (-\Delta I(x,y,z))^{\lambda}&\quad \text{if}\,\Delta I(x,y,z)<0\\ 0 &\quad \text{otherwise} \end{array} \right., \end{array} $$

where the exponent *λ*>0 is the weighting parameter that comprises the fuzzy set [31]. As shown in Table 1, we empirically select *α*=0.4 and *λ*=5 to achieve the best performance in our experiment.

**Table 1:** Directly taking the initial LPM, i.e., up to Section “Step 2): Initial estimation of LPM” of the proposed method, for performance evaluation, under different *α* and *λ*

*α*	*λ*	Accuracy	Precision	Recall	Dice
0.2	1	0.953±0.012	0.321±0.195	0.784±0.165	0.430±0.166
	3	0.959±0.012	0.392±0.198	0.726±0.164	0.478±0.168
	5	0.961±0.011	0.427±0.199	0.694±0.156	0.497±0.165
	7	0.963±0.011	0.446±0.199	0.673±0.158	0.508±0.164
0.4	1	0.965±0.011	0.502±0.199	0.639±0.154	0.532±0.170
	3	0.968±0.012	0.552±0.198	0.582±0.153	0.547±0.169
	5	0.964±0.012	0.555±0.196	0.589±0.151	0.548±0.168
	7	0.940±0.011	0.553±0.201	0.601±0.151	0.547±0.168
0.6	1	0.967±0.012	0.547±0.198	0.596±0.155	0.545±0.170
	3	0.954±0.012	0.553±0.200	0.591±0.154	0.547±0.174
	5	0.940±0.011	0.559±0.203	0.592±0.159	0.546±0.169
	7	0.941±0.011	0.572±0.203	0.574±0.158	0.541±0.165
0.8	1	0.967±0.011	0.549±0.207	0.593±0.154	0.546±0.169
	3	0.940±0.011	0.559±0.204	0.596±0.155	0.546±0.173
	5	0.941±0.011	0.584±0.201	0.557±0.151	0.543±0.172
	7	0.941±0.012	0.623±0.198	0.500±0.151	0.503±0.168

#### Step 3): Amendment to normalization-segmentation template tissue maps

As shown in Fig. 1, the initial LPM estimated based only on voxel intensity is inaccurate – some healthy tissues may show high probability in LPM. To alleviate this problem, we further derive the probability maps of the non-lesion tissues, such as GM, WM, and external CSF of the patient. Typically, these tissue maps can be derived by applying USN pipeline [30]. However, this typical routine requires the input MRI to be from a healthy control subject, as the template does not include a lesion probability map. In this section, we propose a method to amend the templates by introducing an additional lesion probability map - based on the initial LPM – for each patient MRI. Particularly, we amend the templates of GM, WM, and external CSF probability map and the initial LPM. Note that this amendment is conducted independently for each patient image.

Let $P_{\textit {GM}}^{T}(x,y,z)$, $P_{\textit {WM}}^{T}(x,y,z)$, $P_{\textit {eCSF}}^{T}(x,y,z)$ be the template probability maps of GM, WM and external CSF respectively – we do not amend the template probability maps of soft tissue, bone, and air because they are usually not affected by lesion. The basic idea of the amendment is to reduce the template probability value of GM, WM, external CSF at voxels with high lesion probability in terms of the initial LPM estimated in Step 2) and introduce a new template probability map ${P_{L}^{T}}(x,y,z)$ for the lesion. Specifically, at a voxel (*x,y,z*), if *P*_*L*_(*x,y,z*)>*γ*, we simply set $P_{\textit {GM}}^{T}(x,y,z)$, $P_{\textit {WM}}^{T}(x,y,z)$, $P_{\textit {eCSF}}^{T}(x,y,z)$ all to be zero and the new template lesion probability ${P_{L}^{T}}(x,y,z)=1$. Otherwise (i.e., *P*_*L*_(*x,y,z*)≤*γ*), we update the template probability maps by

(4) $$ \begin{aligned} P_{GM}^{T}(x,y,z)&\leftarrow\left(1-\frac{P_{L}(x,y,z)}{\gamma}\right)\cdot P_{GM}^{T}(x,y,z)\\ P_{WM}^{T}(x,y,z)&\leftarrow\left(1-\frac{P_{L}(x,y,z)}{\gamma}\right)\cdot P_{WM}^{T}(x,y,z)\\ P_{eCSF}^{T}(x,y,z)&\leftarrow\left(1-\frac{P_{L}(x,y,z)}{\gamma}\right)\cdot P_{eCSF}^{T}(x,y,z)\\ &{P_{L}^{T}}(x,y,z)\leftarrow\frac{P_{L}(x,y,z)}{\gamma}, \end{aligned}  $$

where *γ* indicates the confidence to the initial LPM – the smaller the value of *γ*, the more confident to the initial LPM *P*_*L*_(*x,y,z*). In this paper, as shown in Section “Results”, we empirically select *γ* with the highest Dice coefficient score, $\gamma =\frac {5}{6}$. Figure 1 shows the amended template probability maps for a patient image.

#### Step 4): LPM refinement

**Fig. 4:**
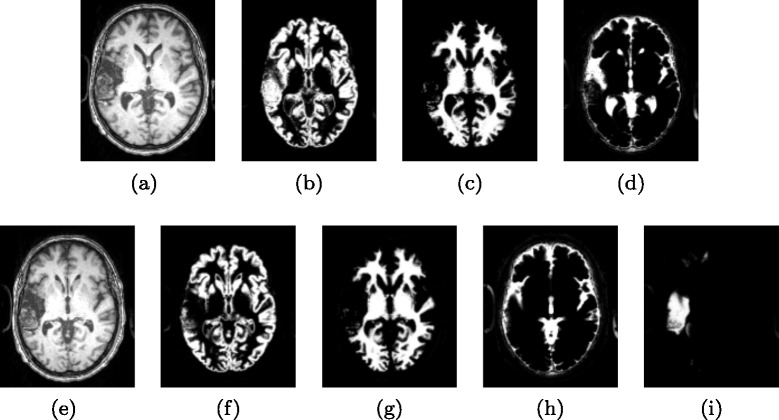
Probability maps of main tissues (and lesions) derived using original SPM templates and the amended templates. (**a**) Normalized patient image without considering lesion using original SPM template. (**b**-**d**) Probability maps of GM, WM, and CSF derived by using original templates. (**e**) Normalized patient image using amended template. (**f**-**i**) Probability maps of GM, WM, external CSF, and lesions derived by using the amended templates

Based on the amended template, we apply the USN pipeline [30, 41] to process the corresponding patient image. This way, we can obtain the probability maps of GM, WM, external CSF and lesion for this patient and we denote them by *P*_*GM*_(*x,y,z*), *P*_*WM*_(*x,y,z*), *P*_*eCSF*_(*x,y,z*), and *P*_*L*_(*x,y,z*) respectively. We further apply a Gaussian smoothing using a kernel with FWHM of 8 *mm* to help reduce the noise in the lesion probability map. A sample result of these probability maps is shown in Fig. 4, where the amended template leads to better-estimated probability maps than the original template. In this step, we also follow the pipeline to derive *P*_*V*_(*x,y,z*), the probability map of ventricle, which is part of the CSF. Later, after both unsupervised and supervised components, we will use the derived *P*_*V*_(*x,y,z*) to exclude ventricle.

### Supervised component

In the supervised components, we take a set of training images of the patients, where experts have manually labeled what we defined as the “ground-truth” for brain lesions. We then extract image and structure features from these training images and use them to train two-class classifiers where each voxel is recognized as either lesion or non-lesion tissues. With the trained classifiers, we can extract the same features from new test images and classify their voxels to get the final lesion detection. Please note that both training images and testing images were acquired using the same MR scanner.

In the feature extraction, we take advantage of both its T1-MRI intensity information, the probability maps of the each brain tissue and lesion derived in the unsupervised component. Specifically, based on the 5×5×5 sliding blocks in the normalized T1-MRI, the probability maps of GM, WM, external CSF and lesion (LPM), we extract three types of features. Based on each of these three types of features, we train three linear kernel SVM classifiers for lesion detection. We combine these three SVM classifiers to achieve the lesion detection. Finally, we automatically select an optimal threshold *t*^∗^ to the SVM output (taking values in [−1,1]) for the binary lesion classification. We will discuss the selection of optimal threshold in Section “Experiment results on in-house dataset and analysis”.

#### Step 5): Feature extraction

To capture the subtle differences between lesion and healthy tissues, at each voxel we consider a 5×5×5 sliding block centered at this voxel for extracting the features of this voxel. For each voxel, features are extracted from five equal-size maps – the T1 image, and the probability maps of GM, WM, external CSF, and lesions (LPM) independently. Specifically, we extract three types of features: 1) zero-order features, 2) 1st-order features, and 3) 2nd-order features and use each type of features for training a separate classifier, as illustrated in Fig. 2.

**Fig. 5:**
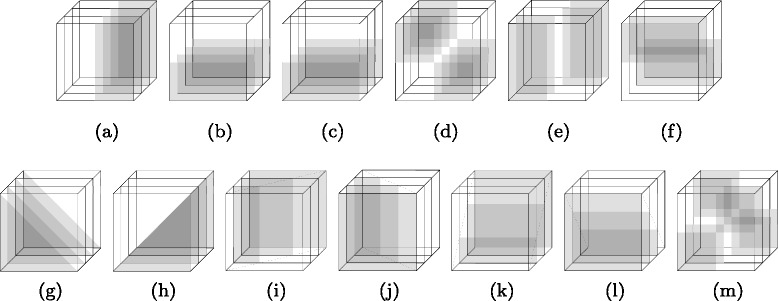
Calculation of 13 3D Haar-like features. (**a**-**f**) are the vertical/horizontal features, (**g**-**m**) are diagonal features. They only compare sums of the same regions in temporal coordinate. Such features are used to describe the lesion edge information
- **1st-order feature:** At voxel (*x,y,z*),we extract its 1st-order features as
  (5) $$\begin{array}{@{}rcl@{}} {}\textbf{f}_{1}(x,y,z)=\frac{1}{27}\sum\limits_{i=-1}^{1}\sum\limits_{j=-1}^{1}\sum\limits_{k=-1}^{1}\textbf{f}_{0}(x+i,y+j,z+k). \end{array} $$
  The 1st-order feature is also a 690-dimensional feature vector.
- **2nd-order feature:** At voxel (*x,y,z*), we extract its 2nd-order features as
  (6) $$\begin{array}{@{}rcl@{}} {}\textbf{f}_{2}(x,y,z)&=&\left(\textbf{f}_{0}(x,y,z)-\textbf{f}_{1}(x,y,z)\right)\\&&\times\left(\textbf{f}_{0}(x,y,z)-\textbf{f}_{1}(x,y,z)\right)^{T}, \end{array} $$

- **Zero-order features:** At each voxel, the corresponding 5×5×5 block provides a 125-dimensional feature vector in each of the five maps, where each dimension takes the *value* (intensity in the T1 image and probability value in the other four maps) of the corresponding voxel in the block. In this block, we further calculate 13 Haar-like features [49] as shown in Fig. 5, where each feature dimension is the average voxel-value difference between the black and white regions within the 5×5×5 sliding block. By concatenating voxel features and Haar-like features from all five maps, the zero-order features have a total dimension of (125+13)×5=690 and we denote it as **f**_0_(*x,y,z*).

which is a 690×690=476,100-dimensional feature vector. Compared with the traditional zero-order features, the 1st-order features are less sensitive to false-positive detection of the lesions and the 2nd-order features emphasize the lesion boundary features by taking the local covariance of the zero-order features, as illustrated in Fig. 2.

#### Step 6): Individual SVM classifiers

SVM [50] is one of the most popular machine-learning methods used in neuroimaging applications for lesion detection [26–29]. It is a kernel-based machine-learning method designed to construct a hyper-plane as the decision plane, which separates two different classes with the largest margin [50]. The use of SVM consists of two phases: training and testing. During the training phase, the algorithm finds statistical properties in the training data that discriminate between healthy tissues and lesion tissues. After the training, during the test phase, the algorithm can classify the lesions in a test data.

In this paper, for each of the above three types of features, we train a linear SVM classifier that estimates the probability of each voxel to be lesion. Here, we use the linear-kernel SVM for these three individual classifiers. Based on these three trained classifiers, we can estimate three lesion probability maps *p*_0_(*x,y,z*), *p*_1_(*x,y,z*), and *p*_2_(*x,y,z*) for a voxel (*x,y,z*) in a test patient image (normalized to standard space), based on the three types of features respectively. These three probability maps take value in the range of [−1,1].

Note that, besides the benefits of the supervised learning (with manually labeled lesion by one expert, the subtle difference between healthy tissue and lesion are further described), features extracted from the T1-MRI, and the probability maps of GM, WM, external CSF, and lesion (LPM) can increase statistical power on classifying lesion from healthy tissue, as shown in Tables 2 and 3.

**Table 2:** Lesion detection performance after applying each main step of the proposed method

	Accuracy	Precision	Recall	Dice
Initial LPM	0.964±0.012	0.555±0.196	0.589±0.151	0.548±0.168
Unsupervised classification	0.981±0.012	0.665±0.183	0.671±0.140	0.663±0.161
Only zero-order features	0.983±0.011	0.747±0.151	0.646±0.145	0.681±0.143
Only 1st-order features	0.983±0.011	0.754±0.149	0.649±0.142	0.687±0.144
Only 2nd-order Features	0.970±0.012	0.526±0.170	0.641±0.152	0.547±0.160
Combine zero- & 1st-order features	0.983±0.011	0.755±0.147	0.649±0.140	0.694±0.126
Combined classification (all 3 features)	0.983±0.011	0.783±0.143	0.685±0.131	0.731±0.106

**Table 3:** The performance of the unsupervised component only and the performance of the proposed method (combining both unsupervised and supervised components)when using different features for supervised training

Unsupervised classification					
		Accuracy	Precision	Recall	Dice
		0.981±0.012	0.665±0.183	0.671±0.140	0.663±0.161
Supervised classification					
Inputs	Classifiers	Accuracy	Precision	Recall	Dice
T1 MRI	zero-order	0.952±0.015	0.150±0.313	0.402±0.203	0.202±0.302
	1st-order	0.957±0.014	0.162±0.306	0.389±0.241	0.214±0.301
	2nd-order	0.954±0.015	0.144±0.347	0.377±0.272	0.193±0.342
	All combined	0.956±0.014	0.187±0.291	0.421±0.198	0.258±0.291
Prob. maps	zero-order	0.981±0.012	0.696±0.178	0.637±0.146	0.665±0.164
of WM, GM	1st-order	0.981±0.012	0.702±0.177	0.621±0.157	0.659±0.166
external CSF, LPM	2nd-order	0.968±0.013	0.503±0.192	0.609±0.159	0.551±0.173
	All combined	0.983±0.012	0.781±0.144	0.681±0.142	0.728±0.112
T1 MRI & prob.	zero-order	0.983±0.011	0.747±0.151	0.646±0.145	0.681±0.143
maps of WM, GM,	1st-order	0.983±0.011	0.754±0.149	0.649±0.142	0.687±0.144
external CSF, LPM	2nd-order	0.970±0.012	0.526±0.170	0.641±0.152	0.547±0.160
	All combined	0.983±0.011	0.783±0.143	0.685±0.131	0.731±0.106

#### Step 7): Combined classification

In this step, we combine the lesion probability maps *p*_0_, *p*_1_, and *p*_2_ generated in Section “Step 6): Individual SVM classifiers” estimated from three individual classifiers into a unified lesion probability map. Specifically, at voxel (*x,y,z*) of a patient image, the final unified lesion likelihood is computed by

(7) $$\begin{array}{@{}rcl@{}}  p(x,y,z)=\omega_{1}\times p_{0}(x,y,z)+\omega_{2}\times p_{1}(x,y,z)\\+ \,\omega_{3}\times p_{2}(x,y,z)-P_{V}(x,y,z), \end{array} $$

where *ω*_1_, *ω*_2_ and *ω*_3_ are the weights for the likelihood estimated by three individual classifiers and *P*_*V*_ is the ventricle probability map derived in Section “Step 4): LPM refinement”. Table 4 provides the details of choosing parameters *ω*_1_, *ω*_2_ and *ω*_3_. Based on the results, we empirically select *ω*_1_=0.1, *ω*_2_=0.3 and *ω*_3_=0.6. Additionally, as shown in Table 3, the combined classification results increase the accuracy, precision, recall and dice coefficient in comparison to lesion detection results using single type of features. Further detailed discussion will be held in Section “Experiment results on in-house dataset and analysis”. Note that, as shown in Fig. 2, this lesion likelihood map is estimated in the standard space, we perform inverse normalization transform to map this lesion likelihood map back to the original native space. Final binary lesion classification is then achieved by thresholding this lesion likelihood map and the selection of optimal threshold will be discussed in Section “Experiment results on in-house dataset and analysis”.

**Table 4:** Lesion-detection performance using the full version of the proposed method, i.e., up to Section “Step 7): Combined classification”, under different *ω*_1_, *ω*_2_, and *ω*_3_

*ω*_1_	*ω*_2_	*ω*_3_	Accuracy	Precision	Recall	Dice
0.1	0.1	0.8	0.982±0.012	0.785±0.139	0.669±0.138	0.722±0.118
0.1	0.2	0.7	0.983±0.011	0.780±0.144	0.684±0.136	0.728±0.112
0.1	0.3	0.6	0.983±0.011	0.783±0.143	0.685±0.131	0.731±0.106
0.1	0.4	0.5	0.983±0.011	0.789±0.138	0.680±0.133	0.730±0.111
0.1	0.5	0.4	0.983±0.011	0.792±0.137	0.675±0.135	0.729±0.112
0.1	0.6	0.3	0.983±0.011	0.787±0.140	0.675±0.134	0.727±0.113
0.1	0.7	0.2	0.982±0.011	0.787±0.139	0.672±0.135	0.725±0.112
0.1	0.8	0.1	0.983±0.011	0.787±0.141	0.665±0.147	0.721±0.114
0.2	0.1	0.7	0.982±0.011	0.776±0.149	0.685±0.133	0.728±0.112
0.2	0.2	0.6	0.983±0.011	0.783±0.141	0.684±0.133	0.730±0.112
0.2	0.3	0.5	0.983±0.011	0.788±0.138	0.679±0.133	0.729±0.112
0.2	0.4	0.4	0.983±0.011	0.792±0.136	0.673±0.134	0.728±0.112
0.2	0.5	0.3	0.983±0.011	0.788±0.137	0.674±0.134	0.727±0.113
0.2	0.6	0.2	0.983±0.011	0.787±0.137	0.669±0.139	0.723±0.113
0.2	0.7	0.1	0.983±0.011	0.785±0.139	0.666±0.148	0.721±0.114
0.3	0.1	0.6	0.983±0.011	0.782±0.140	0.684±0.135	0.730±0.112
0.3	0.2	0.5	0.983±0.011	0.788±0.136	0.677±0.134	0.728±0.112
0.3	0.3	0.4	0.983±0.011	0.790±0.135	0.673±0.135	0.727±0.112
0.3	0.4	0.3	0.982±0.011	0.787±0.136	0.673±0.137	0.726±0.113
0.3	0.5	0.2	0.983±0.011	0.787±0.135	0.668±0.141	0.723±0.114
0.3	0.6	0.1	0.982±0.011	0.784±0.138	0.665±0.137	0.720±0.114
0.4	0.1	0.5	0.983±0.011	0.786±0.137	0.678±0.133	0.728±0.111
0.4	0.2	0.4	0.983±0.011	0.787±0.137	0.675±0.134	0.727±0.112
0.4	0.3	0.3	0.983±0.011	0.787±0.139	0.672±0.134	0.725±0.112
0.4	0.4	0.2	0.983±0.011	0.786±0.139	0.668±0.141	0.722±0.114
0.4	0.5	0.1	0.983±0.011	0.783±0.140	0.664±0.143	0.719±0.114
0.5	0.1	0.4	0.983±0.011	0.787±0.139	0.674±0.136	0.726±0.113
0.5	0.2	0.3	0.983±0.011	0.785±0.140	0.672±0.137	0.724±0.112
0.5	0.3	0.2	0.983±0.011	0.784±0.141	0.668±0.143	0.721±0.114
0.5	0.4	0.1	0.983±0.011	0.783±0.143	0.662±0.145	0.717±0.117
0.6	0.1	0.3	0.983±0.011	0.783±0.142	0.672±0.137	0.723±0.112
0.6	0.2	0.2	0.983±0.011	0.782±0.142	0.668±0.144	0.721±0.114
0.6	0.3	0.1	0.983±0.011	0.782±0.142	0.662±0.146	0.717±0.120
0.7	0.1	0.2	0.983±0.011	0.781±0.144	0.668±0.142	0.720±0.113
0.7	0.2	0.1	0.983±0.011	0.782±0.144	0.661±0.150	0.716±0.123
0.8	0.1	0.1	0.983±0.011	0.781±0.146	0.660±0.153	0.715±0.129

An alternative way is to combine all three features and train a single SVM classifier. We choose not to do this because the three features are of different orders and substantially different dimensions. Simply combing them and feeding them into a single SVM may make one feature to dominate the others. In particular, the 2nd-order feature has a dimension of 476,100 and both the zero-order feature and 1st-order feature only have dimensions of 690. In such cases, the use of multiple SVMs for different-order features has been shown to be more effective in other applications [51].

To justify the proposed method, in this paper we compare its performance with several other state-of-the-art lesion detection methods. Stamatakis et al. [13] compare the patient brain to a set of normal controls without segmentation for lesion detection. The T1-MRIs of normal controls and patients are normalized to a standard space. After a spatial smoothing, they are statistically compared voxel-by-voxel to identify regions outside the normal range established by the controls. Instead of using whole brain, Seghier et al. [11] compare the GM/WM between the patient image and normal controls for lesion detection. This algorithm [11] is performed recursively in Sanjuan et al. [20] for enhancing the lesion detection.

## Results

### Experiment data and setting

For our experiments, we compare the proposed method with state-of-the-art lesion detection methods on: 1) in-house dataset, and 2) MICCAI brain tumor image segmentation (BRATS) challenge 2012 dataset. The in-house data and BRATS-2012 data were produced by different scanners with different image size.

For in-house dataset: MRI was acquired with a 3T Siemens Trio system fitted with a 12-channel head-coil. All subjects were scanned with the same 3D T1-MRI sequence using a MP-RAGE (TFE) sequence: *FOV*=256 *mm*×256 *mm*, 160 sagittal slices, 9-degree flip angle, *TR*=2250 *ms*, *TI*=900 *ms*, *TE*=4.2 *ms*, 1 *mm* resolution image. We collected T1-MRI for 60 stroke patients (29 females and 31 males) with an age mean of 61.6 years and a standard deviation of 12.27 years. The average time post-stroke was 39.86 months with a standard deviation of 49.24 months. For the healthy controls used in the unsupervised component, in total we collected 115 brain images from normal subjects without brain damage using the same hardware and sequence. The average age of healthy controls was 70.2 years with a standard deviation of 10.8 years.

For constructing the ground-truth lesion delineation, all patients were also scanned with a high-resolution T2-MRI, yielding a 1 *mm* isotropic image. This sequence used a 3D SPACE (Sampling Perfection with Application optimized Contrasts by Using different flip angle Evolutions) protocol with the following parameters: *FOV*=256×256 *mm*, 160 sagittal slices, variable flip angle, *TR*=3200 *ms*, *TE*=352 *ms*, using the same slice positioning and angulation as the T1 sequence. All the ground-truth lesions are manually delineated on the T2-MRIs, using the MRIcron software [52], since lesions show better contrast in T2-MRI [53]. We then co-register the T1- and T2-MRI using the routine in SPM8 and map the delineated ground-truth lesion from T2-MRI to the T1-MRI as the ground truth for performance evaluation in the experiments. For the in-house data, lesions on T2-MRI were delineated by three co-authors Guo, Fillmore, and Fridriksson, all with sufficient pathology knowledge and training. All the delineation results were further validated by Fridriksson who is a highly experienced pathologist. As a result, each image only has one ground truth.

For MICCAI BRATS 2012 dataset: Brain tumor image data used in this work were obtained from the MICCAI 2012 Challenge on Multimodal Brain Tumor Segmentation (http://www.imm.dtu.dk/projects/BRATS2012) organized by B. Menze, A. Jakab, S. Bauer, M. Reyes, M. Prastawa, and K. Van Leemput. The challenge database contains full-anonymized images from the following institutions: ETH Zurich, University of Bern, University of Debrecen, and University of Utah. The data consists of multi-contrast MR scans of 30 glioma patients (both low-grade and high-grade, and both with and without resection) along with expert annotations for ‘active tumor’ and ‘edema’. For each patient, T1, T2, FLAIR and post-Gadolinium T1 MR images are available. All volumes were linearly co-registered to the T1 contrast image, skull stripped, and interpolated to 1 *mm* isotropic resolution. Please note that we only use T1-MRI for experiments.

### Evaluation metrics

In this paper, we evaluate the performance of a lesion-detection method using a leave-one-out cross validation strategy. In each testing round, all but one patient images are taken for training while the remaining one is used for testing. All the performance measures are averaged over all the 60 testing rounds. For the evaluation criteria, we follow [11, 13, 20] by using the Accuracy, Precision, Recall and Dice coefficient.

$$\begin{array}{@{}rcl@{}} Accuracy=\frac{TP+TN}{TP+TN+FN+FP}, \end{array} $$

$$\begin{array}{@{}rcl@{}} Precision=\frac{TP}{TP+FP}, \end{array} $$

$$\begin{array}{@{}rcl@{}} Recall=\frac{TP}{TP+FN}, \end{array} $$

$$\begin{array}{@{}rcl@{}} Dice=\frac{2\times TP}{2\times TP+FP+FN}, \end{array} $$

where *TP*, *FP*, and *FN* represent the number of true positives, false positives, and false negatives respectively. For example, a voxel is classified as lesion, but not lesion in the ground truth segmentation, is counted as a false positive. We apply different threshold *t* to the lesion likelihood (from the combined classification, see Eq. (8)) to obtain a two-class classification, with which we can draw curves of the Dice’s coefficient.

**Fig. 6:**
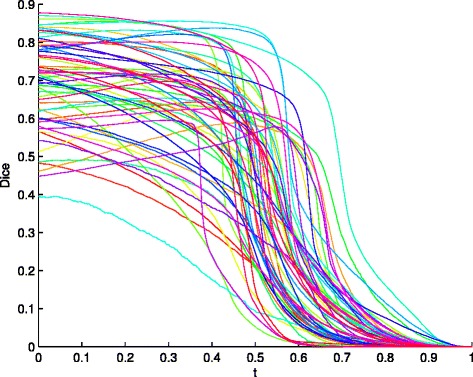
Curves of Dice coefficient (by varying the threshold *t*) for the 60 stroke patients

Figure 6 shows the Dice coefficient at different threshold *t* when testing the proposed method on the 60 patient images – each curve corresponds to one patient (and one round of the leave-one-out testing). We can see that, for most patients, we need to select *t*<0.5 for binary classification to better separate the lesions from the remaining tissues. Without specific claims, in the remainder of the paper all the reported Accuracy, Precision, Recall and Dice coefficient values are obtained by selecting an optimal *t*^∗^ for each MRI that leads to the highest Dice coefficient, for both the proposed method and the comparison methods.

### Experiment results on in-house dataset and analysis

First, we conduct experiments to show the usefulness of each step proposed in this paper. Table 2 shows the performance (averaged over all rounds of the leave-one-out tests) of the proposed method after each main step. Specifically, the initial LPM is the result after Section “Step 2): Initial estimation of LPM” and we threshold this initial LPM, using the optimal threshold *t* for each image that leads to the highest Dice coefficient as described above, to derive the performance after this step. Similarly, the refined LPM is the result in Section “Step 4): LPM refinement” and we use the similar technique to threshold it for a performance evaluation. The rows of ‘Only zero-order features’, ‘Only 1st-order features’ and ‘Only 2nd-order features’ show the performance of the proposed method by using only one of the three types of the statistical features described in Section “Step 5): Feature extraction”, respectively. The Accuracy of the lesion detection is always high (above 90 %) because brain MRIs always contain large areas of dark background outside the brain and these areas can be easily classified correctly as non-lesions. From Table 2, we can see that each main step of the proposed method contributes further performance improvement, in terms of Accuracy, Precision, Recall and Dice coefficient. Additionally, the combination of different order statistical features leads to better lesion-detection performance than using only one type of these features.

In particular, from Table 2, we can see that that the SVM with the 2nd-order feature shows lower performance than the SVMs with the zero-order and 1st-order features in terms of Precision, Recall, Dice and Accuracy. However, if we only combine zero-order and 1st-order features, the Dice coefficient is 0.694±0.126, which is still lower than the combination of all three classifiers (0.731±0.106). This shows that the 2nd-order features still provide complementary information to the other features and help improve the final lesion classification.

Two main free parameters in the initial LPM estimation are *α* in Eq. (2) and *λ* in Eq. (3). Table 1 shows the lesion-detection performance based on the initial LPM after Step 2) under different *α* and *λ*. Based on these results, we select *α*=0.4 and *λ*=5. Table 5 shows the lesion-detection performance based on the refined LPM after Step 4) under different *γ*. Based on these results, we select $\gamma =\frac {5}{6}$. Table 4 shows the lesion-detection performance based on the combined classification after Step 7) under different *ω*_1_, *ω*_2_ and *ω*_3_. Based on these results, we select *ω*_1_=0.1, *ω*_2_=0.3 and *ω*_3_=0.6.

**Table 5:** Directly taking the refined LPM, i.e., up to Section “Step 4): LPM refinement”) of the proposed method, for performance evaluation, under different *γ*

*γ*	Accuracy	Precision	Recall	Dice
$\frac {1}{6}$	0.976±0.012	0.470±0.197	0.611±0.154	0.516±0.178
$\frac {2}{6}$	0.978±0.012	0.510±0.194	0.624±0.151	0.550±0.174
$\frac {3}{6}$	0.979±0.011	0.550±0.189	0.631±0.148	0.582±0.169
$\frac {4}{6}$	0.980±0.011	0.600±0.186	0.634±0.143	0.612±0.166
$\frac {5}{6}$	0.981±0.012	0.665±0.183	0.671±0.140	0.663±0.161
1	0.981±0.012	0.645±0.187	0.590±0.157	0.611±0.166

Second, we conduct experiments to show the effectiveness of combining both unsupervised and supervised components. From Table 3, we can see that, if we directly feed the MRI intensity into the supervised classification, the lesion detection performance is very poor. On the other hand, if we feed the probability maps of brain tissues and lesions estimated from the unsupervised component, i.e., the results after Step 4) in Section “Step 4): LPM refinement”, into the supervised classification, we achieve substantially improved performance. If we combine both intensity and probability map features, we achieve further performance improvements. This shows that the combination of both unsupervised and supervised components boosts the lesion detection performance. In this experiment, we also try the use of each type of the features, i.e., zero-order, 1st-order and 2nd-order statistical features, and the results in Table 3 further confirms that the combination of all three types of features leads to better performance than using only one of them. We also perform a paired-sample one tailed *t*-test to compare lesion detection results after the unsupervised and supervised components. Based on their Dice coefficients, the one-tailed *p*-value for this *t*-test is *p*=6.4*e*−11, and *t*=8.19. This shows that there is a significant performance improvement by including the supervised component.

**Fig. 7:**
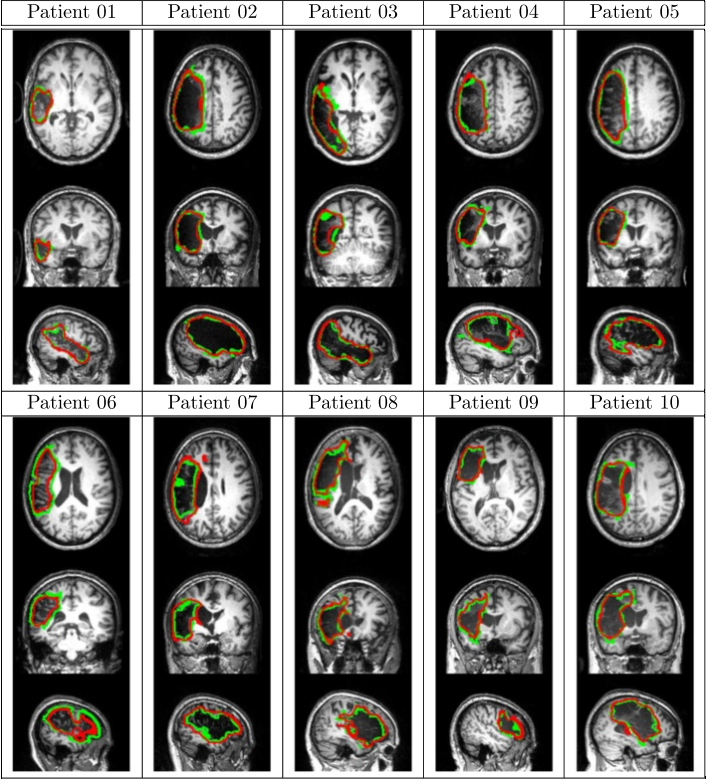
Sample results of lesion detection on selected 2D slices of different patients. Red and green contours indicate the detected lesion boundaries and the ground-truth lesion boundaries, respectively

**Fig. 8:**
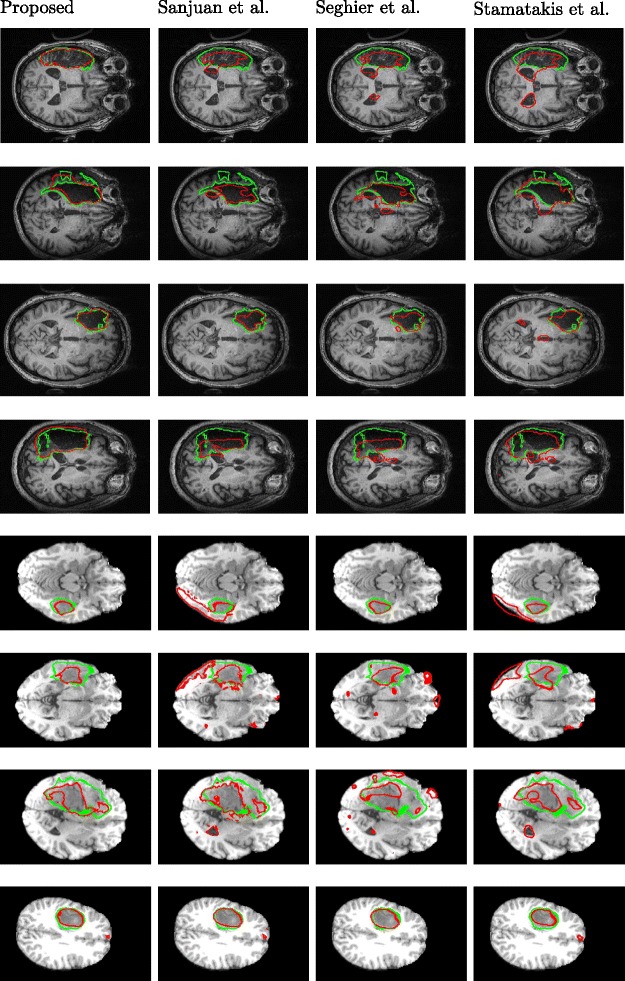
Sample results of lesion detection using the proposed method and the three comparison methods on selected 2D slices. Red and green contours indicate the detected lesion boundaries and the ground-truth lesion boundaries, respectively. Columns 1–4 are the lesion-detection results from the proposed method, Sanjuan et al., Seghier et al., and Stamatakis et al. respectively. Rows 1–4 are the lesion-detection results from in-house dataset. Rows 5–8 are the lesion detection results from MICCAI BRATS 2012 dataset

Finally, we compare the performance of the proposed method with three state-of-the-art lesion detection methods [11, 13, 20] on our collected 60 patient images. From Table 6a, we can see that, the proposed method achieves a substantially better lesion detection performance than these three comparison methods in terms of all the four evaluation criteria, each of which is the average over all 60 rounds of leave-one-out tests. Figure 7 qualitatively shows the detected lesions on selected 2D slices of different patients (from three views), where red contours denote the detected lesion boundaries and the green contours denote the ground-truth lesion boundaries. Figure 8 qualitatively shows the lesion-detection results produced by the proposed method and the three comparison methods on selected 2D slices, where red and green contours indicate the detected lesion boundaries and the ground-truth lesion boundaries, respectively.

**Table 6:** The performance of the proposed method and the three comparison methods on the in-house data

	Accuracy	Precision	Recall	Dice
(a) Threshold *t* is selected to maximize the Dice coefficients against the ground truth				
Stamatakis et al., 2005	0.954±0.016	0.498±0.179	0.511±0.174	0.504±0.152
Seghier et al., 2008	0.969±0.012	0.568±0.169	0.539±0.153	0.546±0.151
Sanjuan et al. 2013	0.971±0.011	0.616±0.148	0.599±0.133	0.607±0.122
Proposed Method	0.985±0.011	0.783±0.143	0.685±0.131	0.731±0.106
(b) Threshold *t* is selected by following Eq. (8) without using the ground truth for the proposed method,				
while the thresholds for the comparison methods are the ones suggested in their respective papers				
Stamatakis et al., 2005	0.954±0.016	0.478±0.182	0.513±0.177	0.489±0.154
Seghier et al., 2008	0.969±0.012	0.471±0.179	0.542±0.157	0.506±0.153
Sanjuan et al. 2013	0.971±0.011	0.526±0.163	0.604±0.134	0.557±0.131
Proposed Method	0.981±0.011	0.717±0.151	0.707±0.134	0.698±0.118

The performances reported in Table 6a, for both the proposed methods and the comparison methods, are based on the threshold *t* optimized for each test image (corresponding to a round of leave-one-out cross validation) in terms of the Dice coefficient with the ground truth. In practice, we do not have ground truth lesion detection for new test images and cannot find such an optimal threshold *t* for each image. Instead, we propose a simple strategy to select a threshold *t* for each test image such that it maximizes the structural consistency of the detected lesion. Specifically, considering the 2D serial-sections along the horizontal direction from the top to the bottom, denote the lesion detected (using threshold *t*) on slice *i* to be ${R_{i}^{t}}$. We calculate the overlap between neighboring slices as $\frac {|{R_{i}^{t}}\bigcap R_{i+1}^{t}|}{|{R_{i}^{t}}|}$ with |·| to be the area. With this, we select the threshold *t*^∗^ as

(8) $$ t^{*}=\arg\max_{0\leq t \leq 1}\sum\limits_{i,|{R_{i}^{t}}|\neq0}\frac{|{R_{i}^{t}}\bigcap R_{i+1}^{t}|}{|{R_{i}^{t}}|}.  $$

Table 6b shows the performance of the proposed method using this strategy of threshold selection which is independent of the ground truth. For fairer comparison, in this experiment, ground-truth independent thresholds are also used for the three comparison methods when binarizing the lesion likelihood map for lesion regions. For the three comparison methods, the thresholds are chosen to be the ones as suggested in their original papers and their performances are also reported in Table 6b. We can see that while the performance is lower than the one when using the optimal threshold *t* in terms of the ground truth, the proposed strategy of theshold selection still leads to a Dice coefficient of 69.8 *%*, which is much higher than all three comparison methods.

### Experiment results on MICCAI BRATS 2012 dataset

We also test the proposed method on MICCAI BRATS 2012 dataset. The dataset contains 30 glioma patients (both 24 low-grade MRIs and 6 high-grade T1-MRIs) along with expert annotations for “active tumor” and “edema”. As in the in-house dataset, we use leave-one-out cross validation to train and test the proposed method on MICCAI BRATS 2012 dataset. The values of the parameters *α*, *γ*, *λ*, *w*_1_, *w*_2_, and *w*_3_ are the same as those used for the in-house dataset.

Quantitative comparisons are shown in Table 7a and b. Some examples of lesion/tumor detection from the database are given in Fig. 8. Table 7a reports the performance where the threshold *t* is selected to maximize the Dice coefficients against the ground truth. Table 7b reports the performance where the threshold *t* as in Eq. (8) without using the ground truth.

**Table 7:** The performance of the proposed method and the three comparison methods on MICCAI BRATS 2012 data

	Accuracy	Precision	Recall	Dice
(a) Threshold *t* is selected to maximize the Dice coefficients against the ground truth				
Stamatakis et al., 2005	0.987±0.012	0.401±0.217	0.532±0.168	0.457±0.221
Seghier et al., 2008	0.987±0.011	0.623±0.179	0.484±0.151	0.545±0.157
Sanjuan et al. 2013	0.990±0.011	0.696±0.157	0.523±0.130	0.597±0.119
Proposed Method	0.992±0.011	0.830±0.086	0.555±0.131	0.665±0.120
(b) Threshold *t* is selected by following Eq. (8) without using the ground truth for the proposed method,				
while the thresholds for the comparison methods are the ones suggested in their respective papers				
Stamatakis et al., 2005	0.987±0.012	0.395±0.224	0.530±0.174	0.453±0.233
Seghier et al., 2008	0.987±0.011	0.601±0.184	0.485±0.149	0.537±0.163
Sanjuan et al. 2013	0.990±0.011	0.686±0.161	0.524±0.129	0.594±0.124
Proposed Method	0.991±0.011	0.792±0.104	0.559±0.127	0.654±0.123

## Discussion

In this section, we further discuss several issues and limitations of the proposed method.

In Step 1), we assume brain symmetry to construct a rough estimate of a healthy brain underlying the input lesioned brain for enantiomorphic normalization. However, in practice, brain is by nature asymmetric and the assumption of perfect brain symmetry is not held. In this section, we first conduct an experiment to assess the impact of brain asymmetry to the proposed method. Specifically, we assume that the ground-truth lesion mask is known and we use the USN pipeline with cost function masking in SPM8 [42] for brain normalization. We then use this normalization result to replace the proposed enantiomorphic normalization result in the proposed method and the results on the in-house dataset are reported in Table 8. We can see, by using USN, the Dice of the initial LPM increases by 0.047, the Dice of refined LPM (after unsupervised component) increases by 0.014, and the Dice of the final lesion detection increases by only 0.012. These results show that the assumption of the brain symmetry in the proposed method does not introduce too much impact to final performance of the proposed method. Note that, in practice, the ground-truth lesion mask is unknown and it is impossible to achieve such USN.

**Table 8:** The impact of brain assymmetry to the proposed method

	Accuracy	Precision	Recall	Dice
Using the proposed enantiomorphic normalization				
Initial LPM	0.964±0.012	0.555±0.196	0.589±0.151	0.548±0.168
Unsupervised classification	0.981±0.012	0.665±0.183	0.671±0.140	0.663±0.161
Combined classification	0.983±0.011	0.780±0.144	0.684±0.136	0.731±0.106
Using the ground-truth lesion mask for USN				
Initial LPM	0.971±0.012	0.608±0.193	0.587±0.147	0.595±0.163
Unsupervised classification	0.981±0.012	0.691±0.174	0.678±0.138	0.677±0.164
Combined classification	0.985±0.010	0.794±0.141	0.701±0.127	0.743±0.101

Another issue in the proposed method is the selection of the block size for feature extraction. In all the previous experiments, we choose block size of 5×5×5. We further conduct an experiment to examine the lesion-classification performance using different block sizes and the result on the in-house data is shown in Table 9. We can see that the use of block size 5×5×5 leads to a better performance than the use of block size 3×3×3. Furthermore, the use of block size 7×7×7 can further improve a little the performance (from 0.731 to 0.733 in terms of Dice coefficient). However, we found that the use of 7×7×7 block size takes much more computation time, because of the substantial increase of the feature dimensions. The most time consuming step is to generate the different order features. When increasing the block size from 5×5×5 to 7×7×7, the CPU time to generate features on an image from the in-house data will increase from 133 *min* to 388 *min* on a MAC 10.10.5 computer with a 2.2GHz Intel Core i7 CPU and 16GB memory.

**Table 9:** Lesion detection performance using different block size for feature extraction in Section “Step 5): Feature extraction”

Block size	Accuracy	Precision	Recall	Dice
3 × 3 ×3	0.983±0.011	0.722±0.153	0.683±0.133	0.701±0.112
5 ×5×5	0.983±0.011	0.783±0.143	0.684±0.131	0.731±0.106
7 ×7×7	0.984±0.011	0.785±0.142	0.691±0.127	0.733±0.106

**Fig. 9:**
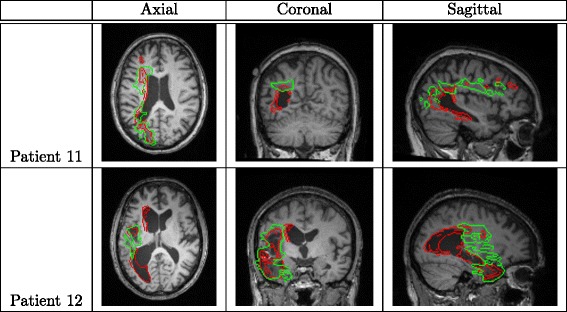
Two failure cases of lesion detection using the proposed method. Red and green contours indicate the detected and ground-truth lesion boundaries, respectively

The presence of brain atrophy will further break the symmetry of the brain and increase the difficulty of brain normalization. The proposed method assumes the brain symmetry for brain normalization and initial lesion estimation. Brain normalization was also important for accurate tissue segmentation, which provides the input to the supervised component. Therefore, the presence of brain atrophy may seriously affect the performance of the final lesion classification. An example is shown in the first row (patient 11) of Fig. 9, where ventricle of the patient is dilated and twisted – the structures surrounding the left occipital lobe is warped towards its counterparts on the right. For this patient, the proposed method only achieves a lesion classification with a Dice coefficient of 0.491.

Partial volume effect changes the voxel intensity and makes it more difficult to capture the tissue boundaries in the segmentation. The proposed method takes tissue segmentation for feature extraction and supervised classification. Therefore, partial volume effect may also affect the performance of the proposed method. Many methods and tools have been developed to identify and correct the partial volume effect [43, 54]. Using these methods and tools to preprocess the images may help reduce its effect to the proposed method.

Another issue may fail the proposed method is that SPM may confuse lesion and CSF since they share similar intensities. But in most cases, this is not a serious issue when lesion is in GM and WM and can be separated with CSF in the normalized standard space. However, when lesion is near CSF or ventricle is substantially deformed, SPM may have troubles in distinguishing the lesion and the CSF. As a result, the proposed method may confuse the lesion and CSF in the classification. An example is shown in the row 2 (patient 12) of Fig. 9, where part of the left lateral ventricle adjacent to the lesion is misclassified as the lesion by the proposed method. For this patient, the proposed method achieves a Dice coefficient of 0.532.

**Fig. 10:**
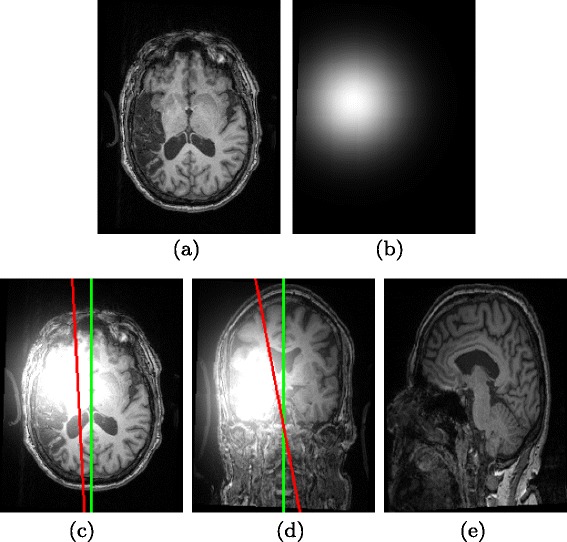
The impact of bias field inhomogeneity to the symmetry axis estimation. (**a**) Original T1-MRI. (**b**) Simulated bias field. (**c**-**e**) samples slices from the simulated T1-MRI with bias field, where red and green lines indicate the detected and ground-truth symmetry axes

Bias field inhomogeneity may fail the proposed method because it will affect the correct identification of the lesioned hemisphere. In addition, the symmetric axis estimation is very sensitive to the bias field inhomogeneity. An example is shown in Fig. 10, where an artificial bias field is added to each slice of a T1-MRI, following the strategies in [55, 56]. Let *X* and *Y* be the size of row and column. Centered at $\left (\frac {X}{10},\frac {Y}{10}\right)$, the added bias field at each 2D slice is a 2D Gaussian with 40 *mm* FWHM and the center intensity of *I*_*B*_ is 10 times of the maximum intensity in the original 3D MRI. Following [55, 56], we first evaluate the estimated symmetry axis against the ground truth axis using the yaw and roll angles between them. Without the added bias field, the yaw and roll angles are 1.788±0.137 and 2.281±0.244 respectively over all the in-house data. With the added bias field, the yaw and roll angles are 2.211±0.154 and 14.899±4.647, respectively, which are much larger than those without the added bias field.

Another failure case of the proposed method is when both hemispheres contain lesions. In this case, the underlying healthy brain cannot be estimated using a hemisphere for brain normalization in Step 1).

## Conclusions

In this paper, we introduced a novel automated procedure for lesion detection from T1-weighted MRIs by combining both an unsupervised and a supervised component. In the unsupervised component, we developed new approaches to identify the lesioned hemisphere and used it to help normalize the patient MRI with lesions and initialize/refine a lesion probability map. In the supervised component, we combined different-order statistical features extracted from both the tissue/lesion probability maps obtained from the unsupervised component and the original MRI intensity and applied three SVM classifiers for final voxel-based lesion classification. In the experiments, we evaluated each main step of the proposed method and verified its effectiveness. Lesion detection on a set of 60 stroke patient MRIs showed that the proposed method could achieve superior performance compared to three state-of-the-art lesion detection methods in terms of four different evaluation criteria. In particular, using the leave-one-out cross validation, the proposed method achieved an average Dice coefficient of 73.1 *%* on the 60 patient MRIs against the ground truth lesions that were manually labeled by trained neurologists. Also, we used the proposed method to detect brain tumor on 30 real tumor T1-MRIs on MICCAI BRATS 2012 dataset and the proposed method achieved an average Dice coefficient of 66.5 *%*.

## Abbreviations

SVM: Support vector machine
MRI: Magnetic resonance imaging
LPM: Lesion probability map
GM: Gray matter
WM: White matter
CSF: Cerebrospinal fluid
FCP: Fuzzy clustering pipeline
SPM: Statistical parametric mapping
